# Practical Notes and Formulæ

**Published:** 1878-06-20

**Authors:** 


### PRACTCAL NOTES AND FORMULÆ.

### TREATMENT OF ALCOHOLISM.

At the New York State Inebriate Asylum, Dr. David H. Kitchen, the superintendent, gives the following as some of the prescriptions which have proved to be most reliable:

R.—Acidi phosphoric dil.................. *m* x.
Elixir calisayæ...................... oz. ss.

M. Sig.—To be taken at one dose, and repeated before each meal.

R.—Tincturæ nucis vomicæ............... gtt. x.

Tincturæ cinchonæ co.,............... oz. ij.

M. Sig.—To be taken in water before meals.

R.—Spt. ætheris sulph. co................ oz. j.

Tincturæ nucis vomicæ............... gtt. x.

M. Sig.—To be taken when the patient suffers from great restlessness.

R.—Extract hyoscyami, fld............... *m* xx.
Chloral hydrat......................... grs. xx.
Aquæ................................. q. s.
M. Sig.—For insomnia.

R.—Extract fluid hyoscyami............ *m*. xxx.
Sig.—In insomnia, and repeat if necessary.

R.—Extract fluid conii.................. *m*. xxx.

Potass. bromidi...................... grs. xx.

Aquæ................................. q. s.

M. Sig.—Repeat in cases of insomnia.

R.—Potass bromidi,
Sodii bromidi, aa .................. grs. xx.
Aquæ................................ q. s.
. Sig.—To be repeated, if necessary, in cases of insomnia; particularly useful where there is marked restlessness.

It has frequently been observed in this institution, that a single glass of milk, taken at bed-time, will produce the same effect as an anodyne or hypnotic, and as a rule we adopt this course before we prescribe medicine; often we prescribe medicine in milk. Experience has also demonstrated that the hot bath does more to relieve the unsettled condition of the nervous system than any medicine we can prescribe.—*Med. and Surg. Rep.*

### EARLY RUPTURE OF MEMBRANES.

Dr. O. E. Newton, of Cincinnati, O., writes:

*Editor Record:*

DEAR SIR—In the April number of your journal, I find some excellent suggestions from Dr. Posey upon the subject of early rupture of the membranes, which article, though good, does not go half far enough, in my opinion.

Thirty years' practice has convinced me that the majority of tedious labors is the result of an over-apprehension against premature rupture of the membranes. It has been my custom to keep ready a sharpened lead pencil, with which I rupture the membranes as soon as the head of the child passes low enough to define it to be in the inferior strait, and there be water enough in front to allow sufficient distention to permit the finger to discover the least bag or fullness in front of the head. When the waters pass, such cases, almost without an exception—all things being right—assume a more progressive character; the pains become more rapid and more effectual; in other words, steady and progressive.

Especially is this the case in very many of those slow labors, which are wholly owing to the superabundance of amniotic fluid, preventing the contact of the walls upon the child during uterine contractions. Very many such cases will linger for hours without progress, which will be at once relieved as soon as the walls of the womb are permitted to press upon the child with each uterine contraction.

All persons who show a remarkable size should be thus relieved early—by early rupture. This has been my uniform rule.

### COD-LIVER OIL WITH HYPOPHOSPHITES AND WHISKY.

The following makes a nice and palatable emulsion:

| | |
|---|---|
| Ol. morrhuæ | fl. oz. 4. |
| Pulv. acaciæ | oz. 2. |
| Syrup pruni virg | fl. oz. 2. |
| Spts. frumenti | fl. oz. 1½. |
| Calcii hypophosph, | |
| Sodii hypophosph, aa | dr. 1. |
| Ol. gaultheriæ | m 24. |
| Ol. amygdalæ amar | m 10. |
| Aquæ q. s. ad | fl. oz. 12. |

Dissolve the hypophosphites in the water. Rub the powdered gum arabic with a little of the water to a paste, then add a small quantity of the cod-liver oil, and triturate thoroughly. Again add a little of the water, and some of the oil, alternately, under constant trituration, until they are thoroughly emulsionized. Dissolve the essential oils in the whisky, mix this with the syrup, and incorporate the latter with the emulsion first formed. —*New Remedies.*

### BORACIC ACID OINTMENT.

| | |
|---|---|
| R.—Boracic acid in powder | 1 part. |
| White wax | 1 " |
| Paraffin | 2 " |
| Almond oil | 2 " |

Melt the wax, paraffin and oil with a gentle heat, then add the acid, and continue stirring until it remains of uniform consistence. Before using it should be reduced to a soft mass by rubbing it in a mortar" or it may be slightly warmed. This is a mild antiseptic ointment much used for burns, scalds, etc., in University College Hospital, London.

### FOR DYSMENORRHŒA.

For the excruciating pains often seen in nervous females at the commencement of the catamenial period, the following will be found an excellent remedy:

| | |
|---|---|
| R.—Tinc. gelseminum | |
| Tinc. camphor, | |
| Tinc. opii deodorized, aa | dr. ij. |

M. Dose, thirty drops every two hours until relieved; also, in dysentery, after the operation of Epsom salts.

### TREATMENT OF METRORRHAGIA BY HYPODERMIC INJECTIONS OF ERGOTINE.

M. C. Paul, in the *Bull. de Therap.*, gives the details of 14 cases in which the uterine hemorrhage was arrested in from 5 to 16 minutes by the following solution:

| | |
|---|---|
| Ergotine | 2 grammes. |
| Water, | |
| Glycerin, aa | 15 grammes. |

of which from 1 to 2 grammes were injected. M. Paul concludes that the hypodermic injection of ergotine is the most rapid and the most efficacious means that we have at our disposal in the treatment of metrorrhagia.—*Clinic.*

### NEUTRALIZING CORDIAL.

| | |
|---|---|
| R.—Rhubarb | four ounces. |
| Saffron | one ounce. |
| Cardamon seeds | one ounce. |
| Nutmeg | one ounce. |
| Soda super carb | one ounce. |
| Ess. peppermint | one ounce. |
| Sugar refined | one pound. |
| Brandy and water sufficient to obtain the strength. | |

Dose, one to two teaspoonfuls. Useful in summer complaints, diarrhœa, etc., etc.

### FOR RIGORS.

When about to take a cold or threatened with local congestion or inflammation, as indicated by cold extremeties, rigors, etc., use the following:

| | |
|---|---|
| R.—Pulvis Doveri | grs. xij. |
| Cinchonia | grs. xx. |

M. Make three powders. Go to bed with a hot rock to your feet, and take a powder every hour until relieved. Pneumonia or plurity may be thus aborted in the congestive or initial stage of the disease.

### GOOD INJECTION FOR GONORRHŒA.

| | |
|---|---|
| R.—Bal. copaiba | 5 drachms. |
| White sugar | 2 drachms. |
| Yellow of an egg, | |
| Water, | 8 ounces. |

M. and inject frequently.

### A DURABLE CEMENT.

This cement, which will not only withstand the action of concentrated and dilute acids, but is also refractory against alkaline leys, ether, alcohols, bisulphide of carbon, benzole, and other dissolving substance, consists simply of a mixture of commercial glycerine and finely pulverized litharge. In mixing glycerine and litharge, a paste is obtained which will harden, in from ten to thirty minutes, depending on the larger or smaller amount of litharge taken. With this cement, all metals, and, in fact, all solid bodies, may be fastened to each other, not only in open air, but also under water and other fluids, as it hardens just as quickly and as well there as in air. It can withstand a temperature of 225°, and may therefore be employod in any case where at present oil cement is used. In connecting chemical or technical apparatus that is exposed to chlorine or hydrochloric acid gas, sulphuric acid, vapors of sulphur, nitric acid, and other strongly corrosive fumes, this cement has been found to be excellent. The same may be said about the fumes of alcohol, ether, bisulphide of carbon and carbohydrides in general, which, even boiling, are totally inactive upon it.—*Mich. Med. News.*

### RHEUMATIC TONIC.

The following is Dr. Horton's remedy for rheumatism—well adapted to cases where the disease is associated with indigestion or a debilitated and nervous condition of the system—

| | |
|---|---|
| R.—Tincture of strychnine | oz. j. |
| Tinc. cimicifuga | oz. ij. |
| Muriate of morphine | grs. xij. |

M. Dose, thirty to sixty drops night and morning.

### HOPE'S DYSENTERIC MIXTURE.

| | |
|---|---|
| R.—Nitric acid | 8 drops. |
| Tinc. opinm | 40 drops. |
| Camphor water | 8 ounces. |

M. Dose for an adult, one teaspoonful.

### A GOOD FORMULA FOR THE SUMMER DIARRHŒAS OF CHILDREN.

| | |
|---|---|
| ℞.—Sugar of milk | oz. ss. |
| Pepsin | grs. xl. |
| Lactic acid | gutt. xxx. |
| Hydrochloric acid | gutt. xxx. |
| Tinct. xanthoxlum | drs. j. |
| Water | ozs. j ss. |

M. S. Twenty drops every half hour for a child one year old.—*St. Louis Eclectic.*

### IMPROVED CHALK MIXTURE.

| | |
|---|---|
| R.—Creta. prep. | |
| Sach alb., aa | oz. ss. |
| Glycerin, pure | dr. ij. |
| Cinnamon water | oz. ijss. |
| Creasote | gtts. iij. |

M. Dose, one teaspoonful, to be well shaken. Excellent for acid diarrhœa of children. It will keep indefinitely.

### CARRON OIL IN ANAL FISSURE.

This painful affection, which has heretofore resisted almost all forms of treatment by local applications, has been successfully managed by Carrere, who states in *Annales de la Med. de Grand* that he applies the mixture of lime and water and linseed oil, so commonly used in burns. This is done several times daily, and in all cases he has obtained a cure it at farthest, eight days.—*Alleg. Med. Cent.-Zeit.*

### CHLORAL DANGEROUS IN DELIRIUM TREMENS.

In Nephey's *Medical Therapeutics*, it is said: "It has been shown beyond reasonable doubt, by Dr. Madison March, of Louisiana, and later by Dr. Ernest Magnan, of Paris, that drunkards do not bear chloral at all well. Its use by them, even in moderate doses, is liable to be followed by sudden death."—*Detroit Lancet.*

### DR. ATLEE'S NIPPLE WASH.

| | |
|---|---|
| R.—Pulv. gum Arabac | dr. ss. |
| Biborate of soda | grs. x. |
| Tinc. myrrh | dr. j. |

M. Apply after sucking.

